# The Estimated Effect of Physicians' Advice for Smoking Cessation and Assumed Tobacco Retail Price Increase on Smoker's Intention to Quit in Shanghai, China: A Cross-Sectional Study

**DOI:** 10.3389/fpubh.2021.740476

**Published:** 2021-09-27

**Authors:** Ruiping Wang, Xiangjin Gao, Yan Qiang, Qiong Yang, Xiaopan Li, Bin Li

**Affiliations:** ^1^Shanghai Skin Disease Hospital, Tongji University, Shanghai, China; ^2^College of Public Health, Shanghai University of Traditional Chinese Medicine, Shanghai, China; ^3^Songjiang Fang Song Community Health Service Center, Shanghai, China; ^4^Shanghai Pudong Disease Prevention and Control Center, Shanghai, China

**Keywords:** physicians' advice for quitting, assumed tobacco retail price increase, smoking duration, smoking intensity, current smoker, smoking cessaton intention

## Abstract

**Background:** Tobacco consumption produces a heavy disease burden worldwide, and tobacco price increase, an advertisement for tobacco-induced harm, graphic warning labels on cigarette packages and advice of physicians for quitting are policies that have been proved as effective smoking cessation measures. But evidence on the estimated effect of advice of physicians for quitting and assumed tobacco retail price increase on smoking cessation intention among smokers is still limited in China.

**Methods:** From January to April of 2021, we recruited 664 current smokers in Songjiang district of Shanghai by a multistage sampling design. We implemented a logistic regression analysis to calculate the odds ratio (OR) and 95% confidence interval (CI) to explore how smoking cessation intention would be influenced by the assumed tobacco retail price increase as well as advice of physicians for quitting, and used the paired tabulation method to identify the salient tobacco control measures among smokers as well.

**Results:** A total of 664 current smokers included 548 males (82.53%), with an average smoking duration of 22.50 years (SD: 11.52 years). About 68.79 and 43.67% of current smokers reported smoking cessation intention due to advice of physicians for quitting and the assumed tobacco retail price increase, respectively. Logistic regression analysis indicated that female smokers (OR = 2.85 and 4.55), smokers with previous smoking cessation attempt (OR = 3.71 and 3.07), longer smoking duration (OR = 2.26 and 2.68), lower smoking intensity (OR = 1.82 and 1.69), and heavier tobacco burdens (OR = 1.67 and 2.22) had the higher intention of smoking cessation both due to advice of physicians for quitting and due to assumed tobacco price increase, respectively. Meanwhile, the advice of physicians for quitting was more effective and acceptable (over 80%) than the assumed tobacco price increase for inducing smokers to consider quitting in Shanghai.

**Conclusions:** Smokers have a high intention of smoking cessation in Shanghai, and the advice of physicians for quitting is a potentially more salient tobacco control measure than the assumed tobacco retail price increase. Incorporating smoking duration, intensity, personal burden as well as noncommunicable disease (NCD) status of smokers into the implementation of tobacco control measures is beneficial for descending smoking prevalence.

## Background

Tobacco use is associated with the increased risk of a variety of health problems (1). Generally, tobacco smoking has a negative health effect and is also the single largest preventable cause of morbidity and mortality all over the world (2). Tobacco use causes almost 6 million deaths globally each year and shows a continuing increased trend (3). World Health Organization (WHO) estimates that the number of people addict to tobacco smoking is over 1 billion, and over 8 million people will die from tobacco-related diseases by 2030 if the current trends continue (3, 4). Tobacco use is a recognized risk factor for many chronic diseases, which leads to a heavy burden involving health care and economic as well as social costs in all countries (5). In order to curb the elevated prevalence of tobacco smoking, many public health interventions have been attempted in efforts, including health literacy campaigns, targeted smoking bans, advertise restrictions, and excise taxes, etc (4–6).

Previous studies demonstrate that smoking restrictions, advertisement warnings about the dangers of tobacco use, and graphic warning labels on cigarette packages are all policies that have to be effective for smoking cessation in the world (7–9). Research evidence suggests that smoking cessation advice of physicians is a powerful motivator to encourage quitting (10); a recent systematic review indicates that the brief advice of physicians for tobacco smoke cessation can increase the quitting rate by 3 to 6% (10). Meanwhile, raising tobacco price has been proved as effective measures for reducing smoking rates (11). International Agency for Research on Cancer (12) indicates that declining smoking prevalence and reduced tobacco consumption is strongly associated with the increased costs of tobacco consumption. Researches among US adults also demonstrate that a 10% tobacco price increase results in a 3–5% decrease in cigarette demand (13). So, advice of physicians for smoking cessation and tax-induced cigarette price increase may represent the key policy options to drive smoking cessation among smokers (14).

China is facing a public crisis with the tobacco smoke prevalence of 57% among males and 3% among females, and an estimated 320 million people are smokers with current smoking prevalence. The Global Adult Survey (GATS) conducted in 2010 indicates that 300 million adults in China are current smokers, nearly 1 million smokers die from tobacco-related diseases, and about 52% of non-smokers are exposed to tobacco smoke for at least 15 min daily and over 1 day each week (15–18). Due to the tobacco-induced heavy diseases burden, China signed the WHO Framework Convention on Tobacco Control (FCTC) in 2003 and implemented the FCTC in 2006, but the official assessment report indicates that China has made limited progress toward tobacco control and the current smoking prevalence was still high, especially among the elder male population (15, 19). To reduce the health burden of tobacco smoking, smokers in China need to be encouraged to quit smoking and prevented from smoking (20). However, researches conducted in 1996, 2002, and 2010 in China suggest that the majority of smokers have no intention to quit (20–22), which indicates the long way of tobacco control work in China. So further studies examining intentions to quit smoking among smokers and exploring the salient tobacco control policies are crucial to reduce the smoking prevalence and tobacco-induced diseases burden in China.

In this paper, we conducted a cross-sectional study in Shanghai, China. We aim to understand the smoking intensity, smoking duration, and smoking cessation intention among current smokers, to explore how smoking cessation intention would be influenced by the assumed tobacco retail price increase as well as advice of physicians for quitting, and to evaluate and identify the salient tobacco control measures in Shanghai, China.

## Methods

### Study Population

This study was conducted in Songjiang district, a rural area of Shanghai from January to April 2021. The 664 current smokers in this study don't constitute a random sample of the entire smoking population in Shanghai, but were judiciously selected based on the geographical representation. We employed a multistage sampling design to recruit tobacco smokers among the 15 subdistricts of Songjiang, Shanghai. First, seven subdistricts were randomly selected from the 15 subdistricts. Second, two residential blocks were selected randomly within each of the selected subdistrict. Third, within each selected residential block, a complete list of home addresses of all households was compiled previously, and then, a sample of 100 households was extracted randomly from the list without replacement. Finally, the enumerated 100 households were randomly ordered, and smokers were then approached following the randomized order until 50 current smokers were surveyed. Due to the low prevalence of tobacco smoking in female, one male smoker and one female smoker from each selected household were surveyed whenever possible to increase the sample size of female smoker, and the next birthday (23) method was applied to select the individual to be interviewed whenever there was more than one qualified person in the same household. This study was approved by Review Board of Shanghai Skin Diseases Hospital of Tongji University (No. SSDH-21-004), and an informed consent paper was signed by each participant before the questionnaire interview; finally, 664 smokers [a response rate was 94.86% (664/700)] completed the interview and were finally included in data analysis.

### Data Collection

In this study, the electronic questionnaire for data collection is an Android pad-assisted software, which is convenient to paperless data input and the whole course audio record benefits the subsequent data quality inspection. The questionnaire includes (1) the demographic information (age, gender, education level, marital status, and occupation, etc), (2) history of non-communicable diseases [hypertension, diabetic mellitus, coronary disease, asthma, chronic obstructive pulmonary diseases (COPD), chronic bronchitis, cerebral apoplexy, and cancer], (3) tobacco consumption information (daily tobacco smoking consumption, number of years as a smoker, daily consumed tobacco retail price, quitting history, etc), and (4) smoking cessation intention due to advice of physicians for quitting, the assumed tobacco retail price increase as well as a graphic warning label on cigarette packages.

### Definition and Index Calculation

In this study, we define a smoker as a person who smoked at least 100 cigarettes in a lifetime, and a current smoker as a smoker who still smoke at least once a week at the time of the investigation. We define smoking duration as the time interval (year) between the age at investigation and age at smoking initiation among current smokers, and classify it into three groups (“ <10” years, “10–20” years, and “>20” years); smoking intensity is defined as the number of cigarettes smoked per day and is categorized as <20 cigarette, and 20 cigarettes or more. Personal tobacco burden is defined as the percentage of the monthly expense on tobacco purchase divided by the individual monthly income, and is classified into <20% and equal or over 20%. In this study, age of current smokers is classified into five groups (“18–29,” “30–39,” “40–49,” “50–59,” and “60–79”). Education is recorded as completed years of schooling and categorized into four categories of 1–6 years (primary school), 7–9 years (junior high school), 10–12 years (senior high school), and >12 years (college and above). Individual monthly income is divided into four groups (<5,000 RMB, 5,000–10,000 RMB, 10,001–20,000 RMB, and over 20,000 RMB).

### Data Analysis

We applied SAS software (version 9.2) for data analysis. We describe the data as means and standard deviations (SD) for quantitative variables with normal distribution, and as median and interquartile range (IQR) for quantitative variables with skewed distribution, and we described the data by frequency counts and proportions (rate) for qualitative variables. Student's *t*-test and Mann–Whitney *U*-tests were applied to examine the difference between quantitative variables with normal or skewed distribution, respectively, and a chi-squire test was used to examine the differences between qualitative variables. Logistic regression was applied to calculate the odds ratios (OR) and 95% confidence interval (95% CI) of smoker who had the intention to quit due to advice of physicians for quitting or the assumed tobacco retail price increases, respectively, with the adjustment of potential confounders, which was identified by the directed acyclic graphs (DAGs). Figures were produced to describe the exclusive effect of advice of physicians for quitting or assumed tobacco retail price increases on the intention of smokers to quit, so as to select the salient tobacco control measures among smokers in Shanghai. In this study, a *p*-value of < 0.05 (two-tailed) was considered as statistically significant.

## Results

The 664 current smokers included 548 males (82.53%), with an average age of 43.70 years (SD: 11.42 years). The majority of current smokers were married (84.34%), with an education of college and above (61.75%). Approximately 75% of current smokers had an individual monthly income of over 5,000 RMB, and 21.69% of them had at least one type of non-communicable diseases (NCDs). The prevalence of hypertension, chronic bronchitis, diabetic mellitus, and tumor was 13.86, 6.02, 3.31, and 2.11%, respectively. Table 1 indicates that male smokers were elder, with lower individual monthly income, had higher proportion of married status, and had higher prevalence of NCD, in comparison with female smokers (Table 1).

**Table 1:** The demographic feature and non-communicable diseases (NCD) prevalence among current smokers in Shanghai, China.

**Variables**	**Total smokers (*n* = 664)**	**Male smokers (*n* = 548)**	**Female smokers (*n* = 116)**
Age (years)^†^, (mean, SD)	43.70 (11.42)	44.48 (11.50)	40.07 (10.34)
**Age (years)**^**†**^, ***n*** **(%)**			
18–29	72 (10.84)	50 (9.12)	22 (18.97)
30–39	176 (26.51)	142 (25.91)	34 (29.31)
40–49	218 (32.83)	182 (33.21)	36 (31.03)
50–59	140 (21.08)	120 (21.90)	20 (17.24)
60–79	58 (8.73)	54 (9.85)	4 (3.45)
**Marital status**, ***n*** **(%)**			
Unmarried	52 (7.83)	38 (6.93)	14 (12.07)
Married	560 (84.34)	468 (85.40)	560 (79.31)
Divorced/widow/widower	52 (7.83)	42 (7.66)	52 (8.62)
**Education**, ***n*** **(%)**			
Primary	14 (2.11)	14 (2.55)	0 (0.00)
Junior High	72 (10.84)	64 (11.68)	8 (6.90)
Senior High	168 (25.30)	140 (25.55)	28 (24.14)
College and above	410 (61.75)	330 (60.22)	80 (68.97)
**Individual monthly income (RMB)**, ***n*** **(%)**			
Less than 5, 000	162 (24.40)	124 (22.63)	38 (32.76)
5, 000–10,000	248 (37.35)	208 (37.96)	40 (34.48)
10,001–20,000	180 (27.11)	150 (27.37)	30 (25.86)
Over 20,000	74 (11.14)	66 (12.04)	8 (6.90)
**Residency status**, ***n*** **(%)**			
Local resident	562 (84.64)	466 (85.04)	96 (82.76)
Nonlocal resident	102 (15.36)	82 (14.96)	20 (17.24)
**Non-communicable diseases**^**†**^, ***n*** **(%)**			
At least 1 type	144 (21.69)	136 (24.82)	8 (6.90)
0 type	520 (78.31)	412 (75.18)	108 (93.10)
**Prevalence of noncommunicable diseases**, ***n*** **(%)**			
Hypertension^†^	92 (13.86)	86 (15.69)	6 (5.17)
Chronic bronchitis^†^	40 (6.02)	38 (6.93)	2 (1.72)
Diabetes	22 (3.31)	22 (4.01)	0 (0.00)
Tumor	14 (2.11)	12 (2.19)	2 (1.72)
Chronic obstructive pulmonary diseases	6 (0.90)	6 (1.09)	0 (0.00)
Coronary heart diseases ^♢^	4 (0.60)	4 (0.73)	0 (0.00)
Asthma ^♢^	4 (0.60)	4 (0.73)	0 (0.00)

† *The differences between male smokers and female smokers on demographic feature and prevalence of NCD was statistically significant (P < 0.05)*.

♢ *: for Fisher's exact test*.

### Smoking Intensity, Duration, and Tobacco Consumption Condition

Table 2 demonstrates that the average age of tobacco smoking initiation was 21.21 years (SD: 4.87 years), and the mean value of smoking duration was 22.50 years (SD: 11.52 years). About 664 current smokers consumed 15 cigarettes per day on average (IQR: 10–20 cigarettes) and spent 700 RMB per month on tobacco purchase (IQR: 300–1000 RMB). The prevalence of smoking intensity <20 cigarettes per day was 60.54%, and the prevalence of personal tobacco burden <20% was 55.17%. About 48.19% of current smokers attempted smoking cessation but all relapsed; the median value of smoking cessation attempt times was 2 (IQR: 1–3). Meanwhile, the smoking initiation age of female smokers was smaller than that of male smokers, and female smokers spent more money on tobacco purchase and had a higher prevalence of personnel tobacco burden ≥20% (44.83%), but had a lower proportion of smoking cessation attempt (15.52%) (Table 2).

**Table 2:** The smoking intensity, smoking duration, tobacco expenses, and smoking cessation attempt among current smokers in Shanghai, China.

**Variables**	**Total smokers (*n* = 664)**	**Male smokers (*n* = 548)**	**Female smokers (*n* = 116)**
Age of smoking initiation^†^ (mean, SD)	21.21 (4.87)	21.66 (5.15)	19.05 (2.23)
Years of smoking (mean, SD)	22.50 (11.52)	22.81 (11.65)	21.02 (10.75)
**Smoking duration (years)**, ***n*** **(%)**			
<10	100 (15.06)	80 (14.60)	20 (17.24)
10–20	170 (25.60)	134 (24.45)	36 (31.03)
Over 20	394 (59.34)	334 (60.95)	60 (51.72)
Daily consumed cigarettes on average (median, IQR)	15 (10–20)	15 (10–20)	15 (8–20)
**Smoking intensity (cigarettes/day)**, ***n*** **(%)**			
Less than 20	402 (60.54)	334 (60.95)	68 (58.62)
Equal or over 20	262 (39.46)	214 (39.05)	48 (41.38)
Monthly expense on tobacco purchase (RMB)^†^, (median, IQR)	700 (300–1,000)	600 (300–1,000)	1,000 (1,000–1,000)
**Personal tobacco burden^†^, *n* (%)**			
Less than 20%	508 (55.17)	444 (81.02)	64 (55.17)
Equal or over 20%	156 (44.83)	104 (18.98)	52 (44.83)
**Smoking cessation attempt**^**†**^, ***n*** **(%)**			
Yes	320 (48.19)	302 (55.11)	18 (15.52)
No	344 (51.81)	246 (44.89)	98 (84.48)
Times for smoking cessation attempt, (median, IQR)	2 (1–3)	2 (1–3)	2 (2–7)
**Smoke-free home environment**^**†**^, ***n*** **(%)**			
Yes	492 (74.10)	418 (76.28)	74 (63.79)
No	172 (25.90)	130 (23.72)	42 (36.21)
**Smoke-free workplace environment**^**†**^, ***n*** **(%)**			
Yes	174 (26.20)	124 (22.63)	50 (43.10)
No	490 (73.80)	424 (77.37)	66 (56.90)

† *The differences between male and female current smokers were statistically significant (P < 0.05)*.

### Smoking Cessation Intention Among Current Tobacco Smokers

In this study, 68.79% of tobacco smokers had smoking cessation intention due to advice of physicians for quitting. Smokers aged 50–59 years (84.29%) and with at least one type of NCD (77.78%) had a higher percentage of smoking cessation intention. Meanwhile, the percentage of smoking cessation intention was higher among tobacco smokers with smoking intensity <20 cigarettes per day (73.13%), with personal tobacco burden <20% (74.22%) as well as with previous smoking cessation attempt (81.25%) (Table 3). A logistic regression analysis indicated that female smokers [OR = 2.85, 95% CI:1.68–4.82], smokers aged 50–59 years [OR = 6.36, 95% CI (2.18–18.56)], smokers with previous smoking cessation attempt [OR = 3.71, 95% CI (2.49–5.51)], and with at least one type of NCD [OR = 2.28, 95% CI (1.38–3.77)] had higher intention of smoking cessation due to advice of physicians for quitting. However, smokers with smoking intensity ≥ 20 cigarette per day [OR = 0.55, 95% CI (0.38–0.81)], with tobacco burden <20% [OR = 0.60, 95% CI (0.39–0.92)] had lower intention of smoking cessation due to advice of physicians for quitting (Table 4).

**Table 3:** The smoking cessation intention due to physician advice and assumed tobacco retail price increases among current smokers in Shanghai, China.

**Variables**	**Intention to quit due to physician advice for smoking cessation**		**Intention to quit due to assumed tobacco retail price increase**	
	**Yes (*n* = 458)**	**No (*n* = 206)**	**Yes (*n* = 290)**	**No (*n* = 374)**
**Age (years)**^**†‡**^, ***n*** **(%)**				
18–29	48 (66.67)	24 (33.33)	26 (36.11)	46 (63.89)
30–39	112 (63.64)	64 (36.36)	56 (31.82)	120 (68.18)
40–49	148 (67.89)	70 (32.11)	106 (48.62)	112 (51.38)
50–59	118 (84.29)	22 (15.71)	80 (57.14)	60 (42.86)
60–79	32 (55.17)	26 (44.83)	22 (37.93)	36 (62.07)
**Sex**^**‡**^, ***n*** **(%)**				
Male	376 (68.61)	172 (31.39)	224 (40.88)	328 (59.12)
Female	82 (70.69)	34 (29.31)	66 (56.90)	50 (43.10)
**Marital status**, ***n*** **(%)**				
Unmarried	38 (73.08)	14 (26.92)	24 (46.15)	28 (53.85)
Married	380 (67.86)	180 (32.14)	242 (43.21)	318 (56.79)
Divorced/widow/widower	40 (76.92)	12 (23.08)	24 (46.15)	28 (53.85)
**Education**^**‡**^, ***n*** **(%)**				
Primary	8 (57.14)	6 (42.86)	6 (42.86)	8 (57.14)
Junior High	56 (77.78)	16 (22.22)	40 (55.56)	32 (44.44)
Senior High	112 (66.67)	56 (33.33)	82 (48.81)	86 (51.19)
College and above	282 (68.78)	128 (31.22)	162 (39.51)	248 (60.49)
**Individual monthly income (RMB)**^**‡**^, ***n*** **(%)**				
Less than 5,000	112 (69.14)	50 (30.86)	94 (58.02)	68 (41.98)
5, 000–10,000	162 (65.32)	86 (34.68)	102 (41.13)	146 (58.87)
10, 001–20,000	126 (70.00)	54 (30.00)	72 (40.00)	108 (60.00)
Over 20, 000	58 (78.38)	16 (21.62)	22 (29.73)	52 (70.27)
**Residency status**, ***n*** **(%)**				
Local resident	388 (69.04)	174 (30.96)	240 (42.70)	322 (57.30)
Non-local resident	70 (68.63)	32 (31.37)	50 (49.02)	52 (50.98)
**Non-communicable diseases**^**†**^, ***n*** **(%)**				
At least 1 type	112 (77.78)	32 (22.22)	218 (41.92)	302 (58.08)
0 type	346 (66.54)	174 (33.46)	72 (50.00)	72 (50.00)
**Smoking duration (years)**^**‡**^, ***n*** **(%)**				
<10	70 (70.00)	30 (30.00)	42 (42.00)	58 (58.00)
10–20	114 (67.06)	56 (32.94)	58 (34.12)	112 (65.88)
Over 20	274 (69.54)	120 (30.46)	190 (48.22)	204 (51.78)
**Smoking intensity (cigarettes/day)**^**†‡**^, ***n*** **(%)**				
Less than 20	294 (73.13)	108 (26.87)	190 (47.26)	212 (52.74)
Equal or over 20	164 (62.60)	98 (37.40)	100 (38.17)	162 (61.83)
**Personal tobacco burden**^**†‡**^, ***n*** **(%)**				
Less than 20%	368 (72.44)	140 (27.56)	230 (45.28)	278 (54.72)
Equal or over 20%	90 (57.69)	66 (42.31)	60 (38.46)	96 (61.54)
**Previous smoking cessation attempt**^**†‡**^, ***n*** **(%)**				
Yes	260 (81.25)	60 (18.75)	168 (52.50)	152 (47.50)
No	198 (57.56)	146 (42.44)	122 (35.47)	222 (64.53)
**Smoke-free home environment**^**‡**^, ***n*** **(%)**				
Yes	330 (67.07)	162 (32.93)	200 (40.65)	292 (59.35)
No	128 (74.42)	44 (25.58)	90 (52.33)	82 (47.67)
**Smoke free workplace environment**^**†**^, ***n*** **(%)**				
Yes	104 (59.77)	70 (40.23)	76 (43.68)	98 (56.32)
No	354 (72.24)	136 (27.76)	214 (43.67)	276 (56.33)

† *The percentage of intention to quit due to physical advice for tobacco cessation among different groups of current smokers was statistically significant (P < 0.05)*.

‡ *The percentage of intention to quit due to assumed tobacco retail price increases among different groups of current smokers was statistically significant (P < 0.05)*.

**Table 4:** The estimated smoking cessation intention due to physician advice and assumed tobacco retail price increases among current smokers in Shanghai, China.

**Variables**	**Intention to quit due to physician advice for smoking cessation (LRa)**		**Intention to quit due to assumed tobacco retail price increase (LRb)**	
	**OR (odd ratio)**	**95% CI (confidence interval)**	**OR (odd ratio)**	**95% CI (confidence interval)**
**Age (years)**, ***n*** **(%)**				
18–29	1.00	-	1.00	-
30–39	1.11	0.54–2.30	2.01	0.92–4.42
40–49	2.57	0.99–6.65	***7.09***	***2.67***–***18.79***
50–59	***6.36***	***2.18**–**18.56***	***10.21***	***3.60**–**28.95***
60–79	1.17	0.37–3.67	2.84	0.88–9.15
**Smoking duration (years)**, ***n*** **(%)**				
Less than 10	1.00	–	1.00	–
10–20	1.19	0.61–2.32	1.94	0.98–3.87
Over 20	2.26	0.93–5.47	***2.68***	***1.11**–**6.49***
**Smoking intensity (cigarettes/day)**, ***n*** **(%)**				
Less than 20	1.00	–	1.00	–
Equal or over 20	***0.55***	***0.38**–**0.81***	***0.59***	***0.40**–**0.85***
**Personal tobacco burden**, ***n*** **(%)**				
Less than 20%	***0.60***	***0.39**–**0.92***	***0.45***	***0.28**–**0.73***
Equal or over 20%	1.00	–	1.00	–
**Previous smoking cessation attempt**, ***n*** **(%)**				
Yes	***3.71***	***2.49**–**5.51***	***3.07***	***2.10**–**4.48***
No	1.00	–	1.00	–
**Non-communicable diseases**, ***n*** **(%)**				
At least 1 type	***2.28***	***1.38**–**3.77***	***1.93***	***1.23**–**3.01***
0 type	1.00	–	1.00	–
**Smoke free workplace environment**, ***n*** **(%)**				
Yes	***1.71***	***1.14**–**2.56***	-	-
No	1.00	–	–	–
**Sex**, ***n*** **(%)**				
Male	1.00	–	1.00	–
Female	***2.85***	***1.68**–**4.82***	***4.55***	***2.72**–**7.62***
**Smoke-free home environment**, ***n*** **(%)**				
Yes	–	–	***1.71***	***1.15**–**2.55***
No	–	–	1.00	–
**Individual monthly income (RMB)**, ***n*** **(%)**				
Less than 5,000	–	–	1.00	–
5,000–10,000	–	–	***0.34***	***0.21**–**0.56***
10,001–20,000	–	–	***0.29***	***0.17**–**0.50***
Over 20,000	–	–	***0.16***	***0.08**–**0.33***

*LR(a): Multivariate logistic regression for intention to quit smoking due to physician advice for tobacco smoking cessation [covariates mutually adjusted during the logistic regression were selected by directed acyclic graph (DAG) method]*.

*LR(b): Multivariate logistic regression for intention to quit due to assumed tobacco retail price increase [covariates mutually adjusted during the logistic regression were selected by directed acyclic graph (DAG) method]*.

*The bold values indicate the OR was statistically significant*.

Table 3 indicates that 43.67% of smokers had smoking cessation intention due to the assumed tobacco retail price increase. Female smokers, smokers with the education of junior high school, with monthly income <5,000 RMB, had higher intention to quit smoking. Meanwhile, smokers with smoking duration over 20 years, with smoking intensity <20 cigarettes/day, with personal tobacco burden <20%, and with previous smoking cessation attempt had higher intention to quit. A logistic regression analysis demonstrated that female smokers [OR = 4.55, 95% CI (2.72–7.62)], smokers aged 40–49 years [OR = 7.09, 95% CI (2.67–18.79)] and 50–59 years [OR = 10.21, 95% CI (3.60–28.95)], smokers with at least one type of NCD [OR = 1.93, 95% CI (1.23–3.01)], with previous smoking cessation attempt [OR = 3.07, 95% CI (2.10–4.48)], and with smoking duration over 20 years [OR = 2.68, 95% CI (1.11–6.49)] had higher intention of smoking cessation due to assumed tobacco price increase. However, smokers with smoking intensity ≥ 20 cigarette per day [OR = 0.59, 95% CI (0.40–0.85)], with tobacco burden <20% [OR = 0.45, 95% CI (0.28–0.73)] and with higher individual monthly income (OR = 0.34, 0.29, and 0.16), had lower intention of smoking cessation due to tobacco price increase (Table 4).

### Exclusive Effect of Advice of Physicians for Quitting and Assumed Tobacco Price Increase on Smoking Cessation Intention of Smokers

In this study, smokers might report smoking cessation intention due to advice of physicians for quitting and the assumed tobacco price increase, simultaneously. In order to explore the exclusive effect of advice of physicians for quitting (A) and the assumed tobacco price increase on smoking cessation intention (B), we applied the individual paired tabulation method to classify smokers into four groups (A_1_B_1_, A_1_B_0_, A_0_B_1_, and A_0_B_0_). Of the 664 current smokers, 274 smokers had intention to quit both due to advice of physicians for quitting and assumed tobacco price increase (A_1_B_1_, 41.27%), 184 smokers had intention to quit exclusively due to advice of physicians for quitting (A_1_B_0_, 27.71%), only 16 smokers had intention to quit exclusively due to assumed tobacco price increase (A_0_B_1_, 2.41%), and 190 smokers had no intention to quit (A_0_B_0_, 28.61%).

**Figure 1:**
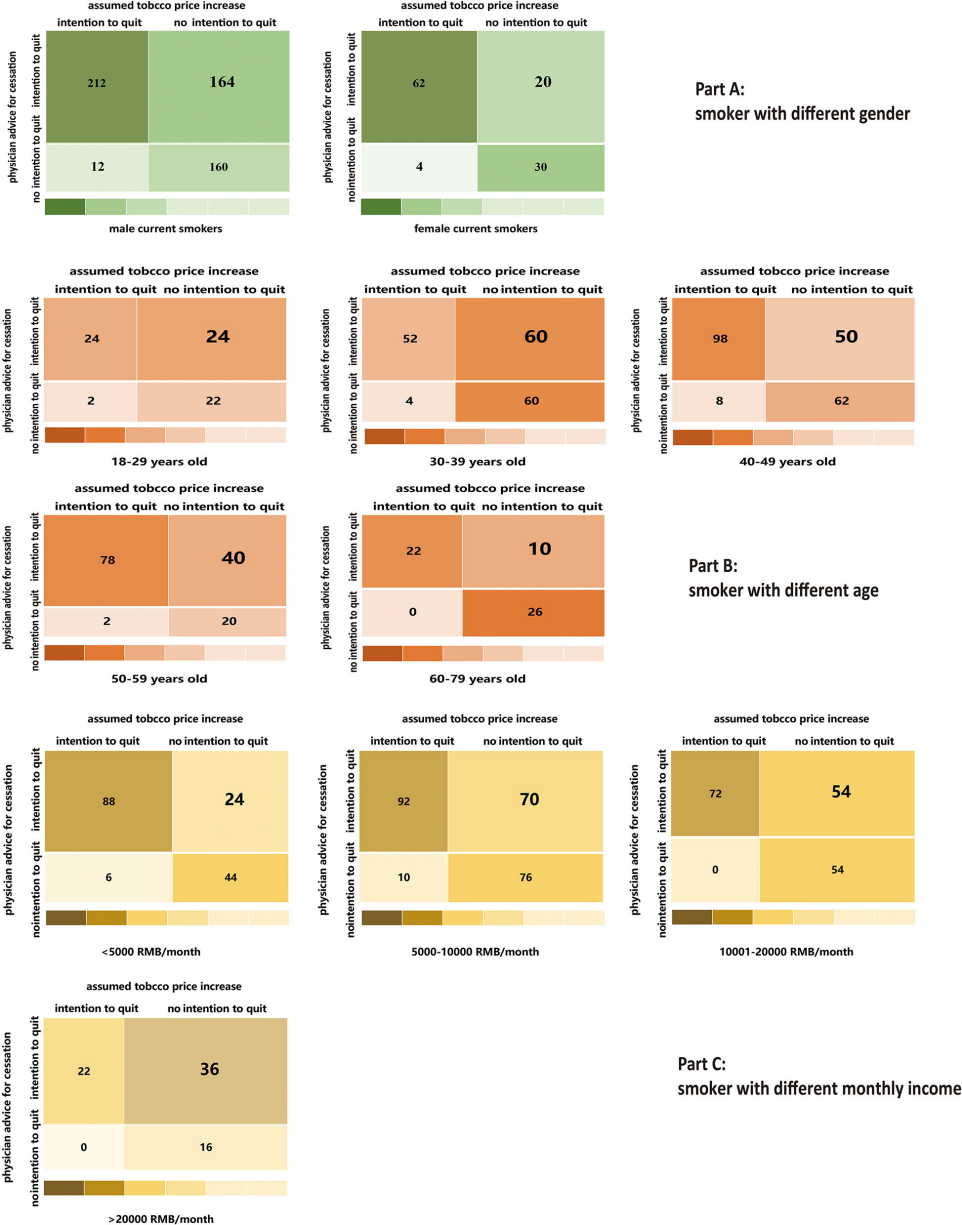
A higher percentage of tobacco smokers reported the intention to quit smoking exclusively due to physicians' advice for quitting than those exclusively due to the assumed tobacco price increase, in spite of their gender **(Part A)**, age **(Part B)** and monthly in come **(Part C)**.

**Figure 2:**
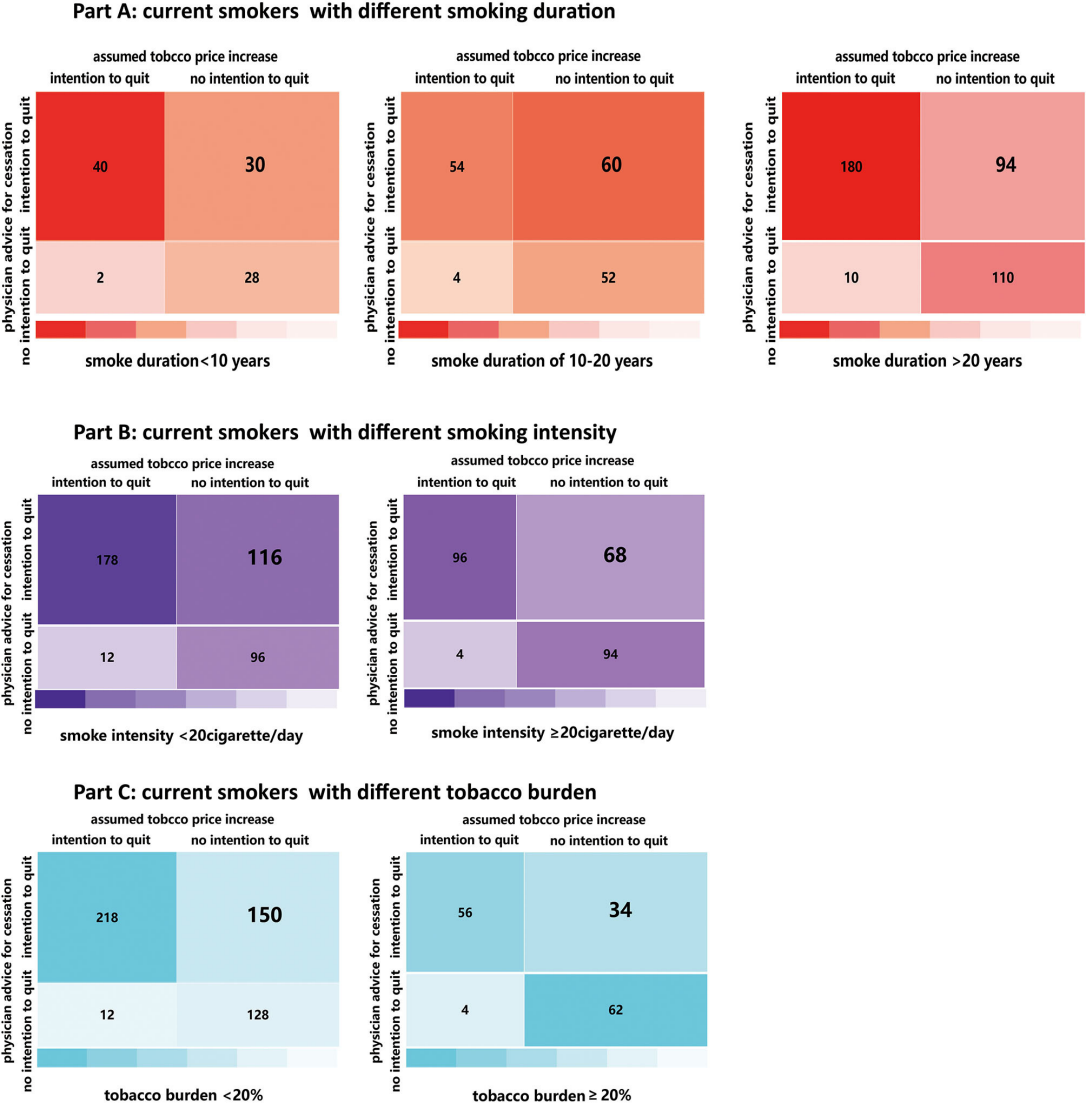
A higher percentage of tobacco smokers reported the intention to quit smoking exclusively due to physicians' advice for quitting than those exclusively due to the assumed tobacco price increase, in spite of their smoking duration **(Part A)**, smoking intensity **(Part B)** and personal tobacco burden **(Part C)**.

**Figure 3:**
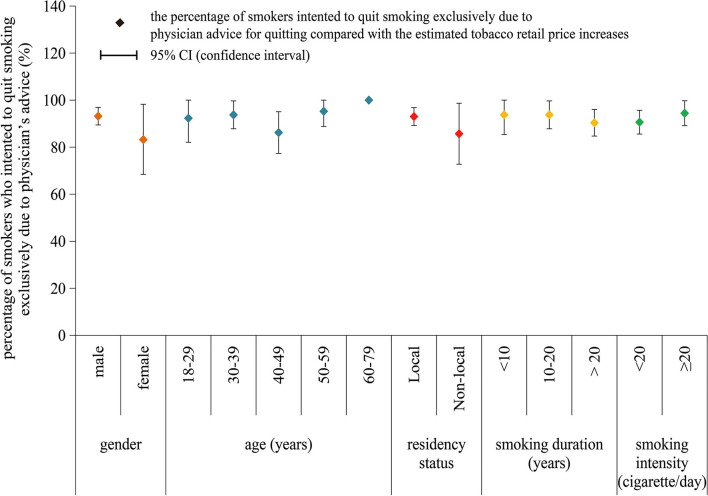
The percentage of smokers who intended to quit smoking exclusive due to advice of physicians for quitting, with the classification of gender, age, residency status, smoking duration, and smoking intensity.

Figures 1, 2 indicate that a higher percentage of tobacco smokers reported the intention to quit smoking exclusively due to advice of physicians for quitting than those exclusively due to the assumed tobacco price increase, in spite of their gender, age, and monthly income, as well as the smoking duration, smoking intensity, and personal tobacco burden. Figure 3 depicts the detailed percentage of smokers who intended to quit smoking exclusively due to advice of physicians compared with the assumed tobacco price increase; the percentages were all over 80% among smokers with difference features (gender, age, residency status, smoking duration, and smoking intensity) (Figures 1–3).

## Discussion

In this study, 68.79 and 43.67% of current smokers in Shanghai reported smoking cessation intention due to advice of physicians for quitting and the assumed tobacco retail price increase, respectively. The percentage of smoking cessation intention among Shanghai current smokers was higher than that in ITC China Survey in 2006 (26%), Jiangxi NCD Survey in 2012 (19%), and Shanghai Tobacco Control Survey in 2013 (20%) (15, 20, 24). The high percentage of smoking cessation intention in this study might attribute to the effect of health education and intervention on smoking cessation in Shanghai (17) and the implementation of new Shanghai Tobacco Control Regulation (enacted in 2016) as well (25). The new Shanghai Tobacco Control Regulation declared that all public place is smoke-free, and all violators, including smoker and the public place owner, would be punished. Meanwhile, the majority of smokers have noticed the physical harmfulness of tobacco use in recent years (24, 25). Another reason for the high smoking cessation intention percentage might be related to the preset scenarios in this study, including advice of physicians for quitting and the assumed tobacco price increases, which made more smokers to consider quitting. However, we should also notice that 48.19% of current smokers in this study had tried to quit smoking with the median attempt times of 2, but all relapsed. So the high percentage of smoking cessation intention was not equal to the high percentage of future actual cessation behavior, so incorporating tobacco control measures covering the repeated intervention and professional counseling contrapuntally would promote the smoking cessation intention transferring into actual cessation behavior.

Previous study in Shanghai demonstrated that 60% of smokers would consider quitting smoking if tobacco retail price doubled (26). This study also indicated that about 43% of current smoker would quit smoking with the assumed tobacco retail price increase. Previous studies showed that smokers who spend a higher proportion of their monthly income on cigarette purchase, with longer smoking duration and smoked less cigarettes per day, had higher intention of smoking cessation (15–26). In this study, we identified that smokers with elder age, longer smoking duration, lower smoking intensity, heavier tobacco burden, at least one type of NCD, and lower individual monthly income had higher intention of smoking cessation, which was in line with the finding from previous studies (27, 28). In China, a long smoking duration predicts elder age, lower monthly income, and higher tobacco burden among smokers, and they are also prone to have chronic diseases problems, makes them sensitive to the assumed tobacco price increase. Lower smoking intensity indicates a mild nicotine dependence among smokers, so they are more likely to change their smoking habit with assumed tobacco price increase than smokers with higher smoking intensity. We recommend that policymakers should adopting excise tax measure to increase the tobacco retail price, and taking tobacco burden, smoking duration, and smoking intensity into consideration for the increases of tobacco price.

Numerous studies indicate that smoking cessation advice of physicians during usual medical consultations is an affordable intervention measure and increases smoking abstinence rates (28). Systematic reviews show that brief intervention of physicians for smoking cessation increases abstinence rates by 47 to 78%, and smoking cessation intention predicts future actual cessation behavior (29, 30). In this study, 68.79% of tobacco smokers reported smoking cessation intention due to advice of physicians for quitting. Moreover, smokers with elder age, longer smoking duration, lower smoking intensity, heavier tobacco burden, and at least one type of NCD had higher intention of smoking cessation. So we recommend that physicians should provide brief advice for smoking cessation during medical consultation and pay more attention to the above-mentioned smokers, which could gradually increase the actual smoking abstinence percentage among smokers.

In this study, we identified that the advice of physicians for quitting was more effective and acceptable than the assumed tobacco price increase for inducing smoker to consider smoking cessation in Shanghai. Previous studies indicated the effectiveness of medical practitioners in facilitating smoking cessation, and the net effect on reducing smoking prevalence could be substantial due to large numbers of physicians and nearly 80% of general population would visit a physician annually (30, 31). Like medical staff in western counties, medical practitioners in China are playing an increasing role in health education and health promotion, and have an increasing array of options to assist smokers who intent to quit (32). So we suggest that the policy makers should consider incorporating the advice of physicians for quitting into tobacco control regulation in the future. Moreover, great efforts should be made to train physicians and health practitioners in providing brief cessation interventions or making a referral to cessation service (33, 34). Nevertheless, the novel optimized “very brief” intervention for only 30 s (35) is an option for physicians with limited counseling time and in busy settings.

This study is the first attempt to estimate how smoking cessation intention would be influenced by the assumed tobacco retail price increase as well as advice of physicians for quitting, and to evaluate and identify the salient tobacco control measures in Shanghai, China. A key strength was that data in this study were collected through face-to-face interviews by an electronic questionnaire, the automated logical check function in the process of data collection and the whole course audio record ensures a better data quality. Considering the lower prevalence of tobacco smoking among the female population in China (about 3%), we applied the special multistage sampling methods to select one male smoker and one female smoker from each selected household were surveyed whenever possible to increase the sample size of female smokers, which in some degree offset the gender disparity of smokers, is another strengthening of this study.

This study has some limitations. First, the sample of smokers doesn't constitute a random sample of the entire smoking population in Shanghai, which limited the generalization of the findings. Second, the use of respondent reports to provide information might induce recall bias. Third, smoking cessation intention with the advice of physicians for quitting and the assumed tobacco retail price increase was only the attitude of smokers but not the actual behavior change, which impeded the observation of the real effect, so implementing an intervention and follow-up study in the future will be a major step forward to evaluate the actual effect of the above-mentioned tobacco control measures. Fourth, we only employed advice of physicians for quitting and the assumed tobacco retail price increase to explore their association with smoking cessation intention among smokers; however, other factors, including tobacco control advertisements, cigarette graphic warning labels as well as tobacco control campaigns such as “the Chinese International Quit and Win Competition,” may also affect the intention of smoking cessation among smokers. The incorporation of some improvements should be considered in further studies.

## Conclusions

Smokers have a high intention of smoking cessation in Shanghai, China, and advice of physicians for quitting is potentially a more salient tobacco control measure than the assumed tobacco retail price increase. Incorporating the smoking duration, smoking intensity, smoking burden as well as NCD status of smokers into the implementation of tobacco control measures is beneficial for descending smoking prevalence.

## Data Availability Statement

The raw data supporting the conclusions of this article will be made available by the authors, without undue reservation.

## Ethics Statement

The studies involving human participants were reviewed and approved by Review Board of Shanghai Skin Diseases Hospital of Tongji University (No. SSDH-21-004). The patients/participants provided their written informed consent to participate in this study.

## Author Contributions

All authors in this paper made substantial contributions to conception and design, acquisition of data, or analysis and interpretation of data, took part in drafting the article or revising it critically for important intellectual content, agreed to submit to the current journal, gave final approval of the version to be published, and agree to be accountable for all aspects of the work.

## Funding

This study was supported by grants from Intelligence Funds of Shanghai Skin Disease Hospital (2021KYQD01), Shanghai Shenkang Hospital Development Center Management Research Program (2020SKMR-32), and the Research Program of Shanghai Sports Bureau (20Q001). The funder had no role in study design, data collection and analysis, decision for publication, or preparation of the manuscript.

## Conflict of Interest

The authors declare that the research was conducted in the absence of any commercial or financial relationships that could be construed as a potential conflict of interest.

## Publisher's Note

All claims expressed in this article are solely those of the authors and do not necessarily represent those of their affiliated organizations, or those of the publisher, the editors and the reviewers. Any product that may be evaluated in this article, or claim that may be made by its manufacturer, is not guaranteed or endorsed by the publisher.

## Abbreviations

WHO: World Health Organization
GATS: global adult tobacco survey
FCTC: WHO framework convention on tobacco control
COPD: chronic obstructive pulmonary disease
OR: odds ratio
CI: confidence interval
DAGs: directed acyclic graphs
SD: standardized deviation
NCD: non-communicable diseases
IQR: interquartile range
ITC: international tobacco control.
